# *Maribacter halichondriae* sp. nov., isolated from the marine sponge *Halichondria panicea,* displays features of a sponge-associated life style

**DOI:** 10.1007/s10482-024-01950-4

**Published:** 2024-03-15

**Authors:** Leon X. Steiner, Jutta Wiese, Tanja Rahn, Erik Borchert, Beate M. Slaby, Ute Hentschel

**Affiliations:** 1 GEOMAR Helmholtz Centre for Ocean Research Kiel, RU Marine Ecology, RD3 Marine Symbioses, Wischhofstraße 1-3, 24148 Kiel, Germany; 2 Christian-Albrechts-University (CAU) of Kiel, Kiel, Germany

**Keywords:** Aminoglycosides, Antibiotic resistance, Baltic Sea, *Halichondria panicea*, *Maribacter*, N-acetyl-β-D-glucosamine utilisation, Sponge-association

## Abstract

A new member of the family *Flavobacteriaceae* (termed Hal144^T^) was isolated from the marine breadcrumb sponge *Halichondria panicea.* Sponge material was collected in 2018 at Schilksee which is located in the Kiel Fjord (Baltic Sea, Germany). Phylogenetic analysis of the full-length Hal144^T^ 16S rRNA gene sequence revealed similarities from 94.3 to 96.6% to the nearest type strains of the genus *Maribacter*. The phylogenetic tree of the 16S rRNA gene sequences depicted a cluster of strain Hal144^T^ with its closest relatives *Maribacter aestuarii* GY20^T^ (96.6%) and *Maribacter thermophilus* HT7-2^T^ (96.3%). Genome phylogeny showed that *Maribacter halichondriae* Hal144^T^ branched from a cluster consisting of *Maribacter arenosus, Maribacter luteus,* and *Maribacter polysiphoniae.* Genome comparisons of strain *Maribacter halichondriae* Hal144^T^ with *Maribacter* sp. type strains exhibited average nucleotide identities in the range of 75–76% and digital DNA-DNA hybridisation values in the range of 13.1–13.4%. Compared to the next related type strains, strain Hal144^T^ revealed unique genomic features such as phosphoenolpyruvate-dependent phosphotransferase system pathway, serine-glyoxylate cycle, lipid A 3-O-deacylase, 3-hexulose-6-phosphate synthase, enrichment of pseudogenes and of genes involved in cell wall and envelope biogenesis, indicating an adaptation to the host. Strain Hal144^T^ was determined to be Gram-negative, mesophilic, strictly aerobic, flexirubin positive, resistant to aminoglycoside antibiotics, and able to utilize N-acetyl-β-D-glucosamine. Optimal growth occurred at 25–30 °C, within a salinity range of 2–6% sea salt, and a pH range between 5 and 8. The major fatty acids identified were C_17:__0_ 3-OH, iso-C_15:__0_, and iso-C_15:1_ G. The DNA G + C content of strain Hal144^T^ was 41.4 mol%. Based on the polyphasic approach, strain Hal144^T^ represents a novel species of the genus *Maribacter*, and we propose the name *Maribacter halichondriae* sp. nov. The type strain is Hal144^T^ (= DSM 114563^T^ = LMG 32744^T^).

## Introduction

The genus *Maribacter* (*Bacteroidota, Flavobacteriia, Flavobacteriales, Flavobacteriaceae*) comprised 30 validly described species and two not yet validated species at the time of writing (Parte et al. 2020). Most species were derived from marine sources such as sponges (Jackson et al. 2015), red and green algae (Hu et al. 2015a; Zhang et al. 2020), seawater (Kang et al. 2018), sediments (Kim et al. 2016), and tidal flats (Lo et al. 2013). Few metabolic features indicating adaptation of members of the genus *Maribacter* to environmental conditions were described. Among them are the production of carbohydrate-active enzymes, such as agarase, alginate lyase, carrageenase, glycoside hydrolases, pectate lyase, polysaccharide lyases, and xylanase being important for habitats, where phytoplankton and macroalgae produce diverse polysaccharides (Martin et al. 2015; Zhan et al. 2017; Wolter et al. 2021). Tolerance to heavy-metals, such as Co^2+^ (10 mM) and Cd^2+^ (0.5 mM) was reported for *Maribacter cobaltidurans* B1^T^, which was isolated from deep-sea sediment (Fang et al. 2017). Only very little is known about the biological role of *Maribacter* sp. strains in host-microbe interactions. *Maribacter* sp. MS6 drives symbiotic interactions with the green macroalga *Ulva mutablis* by releasing morphogenetic compounds, e.g. the hormone–like compound thallusin, which aid in algal morphogenesis, such as rhizoid and cell-wall formation (Kessler et al. 2018; Vallet et al. 2021). A *Maribacter* sp. strain reduces the reproductive success in the diatom *Seminavis robusta* (Cirri et al. 2019). Recently, *Maribacter* sp. strains closely related to *Maribacter dokdonensis* DSW-8^T^ and *Maribacter sedimenticola* KMM 3903^T^ with > 98.50% similarity of 16S rRNA gene sequences were isolated from sponges collected from the Pacific Ocean (Tareen et al. 2022). These *Maribacter* sp. isolates showed antibiotic activity against *Mycobacterium smegmatis*. Three *Maribacter* isolates from the sponge *Hymeniacidon perlevis* sampled at Nord-Pas-de Calais (France) showed antibacterial effects against multi-drug resistant *Staphylococcus aureus*. These isolates were affiliated to *Maribacter arcticus* KOPRI 20941^T^ with approx. 98.50% similarity of 16S rRNA gene sequences (Rodriguez Jimenez et al. 2021). We are currently developing the Baltic Sea sponge *Halichondria panicea* as an experimental model for marine sponge-microbe-phage interactions (Schmittmann et al. 2022). *H. panicea* inhabits coastal areas around the globe and harbors a diverse microbial community including the symbiont *Candidatus* Halichondribacter symbioticus (Knobloch et al. 2019, 2020). Our bacterial cultivation approaches from this sponge species resulted in more than 350 isolates, including 7 *Maribacter* spp. strains. Among them, strain Hal144^T^ attracted our attention as it represents a putatively novel species, serves as a host strain for a novel phage (Steiner et al*.* unpublished), and showed properties related to the host environment. The present study identifies the taxonomic status of strain Hal144^T^ by determining its phylogenetic, physiological, and genomic properties. For the first time, a comparative genome analysis of a *Maribacter* sp. strain and related type strains was performed.

## Materials and methods

### Bacterial isolation and culture conditions

Strain Hal144^T^ was isolated as part of a larger microbial community analysis from the marine breadcrumb sponge *Halichondria panicea*. Sponge individuals were sampled via snorkeling on October 2nd, 2018 from Kiel, Schilksee (Baltic Sea, Germany, coordinates: latitude 54.424705, longitude 10.175133). Specimens were transported in 500 ml Kautex bottles to GEOMAR Helmholtz Centre for Ocean Research Kiel within 2 h after collection. 8.8 g sponge material was rinsed three times with 0.2 µm filtrated and autoclaved Baltic Sea water (BSW) to remove loosely attached particles and microorganisms. The sponge sample was homogenized in a 50 ml Falcon plastic tube, with 35 ml BSW by use of an Ultraturrax for 30 s at 17,500 rpm and serially diluted with BSW from 10^–1^ to 10^–4^. 100 µl of the undiluted suspension and of the dilutions were spread onto a tryptone containing medium (1 g tryptone, 1 g yeast extract, 15 g Bacto-Agar, 1000 ml Baltic Sea water, pH 7.5) and incubated at 25 °C for 7 days. Hal144^T^ was obtained from a colony growing on the dilution 10^–4^ and cultured on tryptone agar plates at 25 °C, then subcultivated using marine medium (MB, 37.4 g BD Difco™ Marine Broth 2216 (Becton Dickinson and Company, New Jersey, USA), 15 g Bacto-Agar, 1000 ml aq. deion.) for 7 days at 25 °C before cryopreservation with the Cryobank System (Mast Diagnostica GmbH, Reinfeld, Germany) at −20 and −80 °C.

### 16S rRNA gene sequencing and phylogenetic analysis

Genomic DNA of strain Hal144^T^ was extracted using the DNeasy Blood & Tissue Kit (Qiagen GmbH, Hilden, Germany) according to the manufacturer’s instructions. The 16S rRNA gene sequence was amplified using the primers Eub27F (5′-GAG TTT GAT CCT GGC TCA G-3′) (Sun et al. 2012) and Univ1492R (5′-GGT TAC CTT GTT ACG ACT T-3′) (Reysenbach et al. 2000) and sequenced via Sanger sequencing (Sanger et al. 1977) at Eurofins Genomics (Ebersberg, Germany) with the primers 534R (Muyzer et al. 1993), 342F (Rainey et al. 1996), and Univ1492R (Reysenbach et al. 2000). The sequenced contigs were assembled and the quality of the sequence was assessed using ChromasPro 2.1.8 (Technelysium Pty Ltd, Brisbane, Australia). The partial 16S rRNA gene sequence comprised 1488 base pairs (bp) and was deposited under the accession number MT406525.2. This PCR-based 16S rRNA gene sequence is identical with the full-length genome-derived sequence (1531 bp) in the overlapping region. The 16S rRNA gene sequences used for phylogenetic analyses were obtained from EzBioCloud 16S database using the featured service “16S_based ID” (Yoon et al. 2017) and compared with the 16S rRNA gene sequence of strain Hal144^T^. This sequence collection was double checked with NCBI (Sayers et al. 2020) using the tool BLAST (Altschul et al. 1990). The full-length 16S rRNA gene sequence of Hal144^T^ was aligned to all *Maribacter* sp. type strains and *Capnocytophaga ochracea* DSM 7271^T^ as the outgroup using the ClustalW tool of MEGA version 11.0.13 (Tamura et al. 2021). Phylogenetic trees were constructed using the Neighbor-Joining (NJ) method (Saitou and Nei 1987) and computing the evolutionary distances with the Maximum Composite Likelihood method (Tamura et al. 2004), the minimum evolution (ME) method in combination with the Maximum Composite Likelihood method and Close-Neighbor-Interchange (CNI) algorithm (Rzhetsky and Nei 1992; Nei and Kumar 2000), and the Maximum-Likelihood (ML) method in combination with the Tamura–Nei model (Tamura and Nei 1993), to ensure the consistency of the tree topology. The phylogenetic trees were constructed in MEGA 11.0.13 (Tamura et al. 2021) by running 1000 bootstrap replications and including 1st + 2nd + 3rd + noncoding positions (Felsenstein 1985). The resulting trees were drawn to scale, with branch lengths measured in the units of the number of base substitutions per site.

### Whole-genome sequencing analysis

Strain Hal144^T^ and *M. aestuarii* JCM 18631^T^ (= GY20^T^), (obtained from RIKEN BioResource Research Center, Tsukuba, Japan), were grown on MB at 25 °C for 7 days. DNA was extracted with Qiagen Genomic-tip 100/G (Hilden, Germany), following the standard protocol by the manufacturer. The extracted DNA had a concentration of 279 ng/µl for Hal144^T^ and 397 ng/µl for JCM 18631^T^. The quality of the DNA met the criteria, i.e. A260/280 ratio of > 1.8 and A260/230 ratio of < 1.8, according to NanoDrop (Thermo Fisher Scientific, Germany) measurements. The genome was sequenced with MinION nanopore technology (Oxford Nanopore Technologies, Oxford, UK) using a MinION Flongle Flow-Cell (Cat.No. FLO-FLG001) with the Flow Cell Priming Kit (Cat.No. EXP-FLP002) and the Rapid Sequencing Kit (Cat.No. SQK-RAD004), following the manufacturer’s protocols. The super-accurate model of Guppy (Oxford Nanopore Technologies plc. Version 6.2.1 + 6588110, dna_r9.4.1_450bps_sup) was used for basecalling of the nanopore reads. Initially, the MinION data were assembled using Miniasm (version 0.3-r179) (Li 2016), then polished with Racon (version 1.5.0) (Vaser et al. 2017) and Medaka (version 1.4.3, model r941_min_sup_g507) (Oxford Nanopore Technologies 2017).

The annotation was prepared using RAST (Aziz et al. 2008), BV-BRC (Olson et al. 2023), KEGG (Kanehisa and Goto 2000), eggNOG (Huerta-Cepas et al. 2019), AntiSMASH (Blin et al. 2021), and PGAP (Tatusova et al. 2016). Bakta v1.7.0 (Schwengers et al. 2021) annotation pipeline was used for gene assignment to cluster of orthologous groups (COG) functional categories with the NCBI COG database v2020 (Galperin et al. 2019). For the comparison across related *Maribacter* sp. genomes, the count of each COG category was normalized with the respective genome size, and expressed as a percentage of the total COG sum (genome content %). COG categories with individual differences larger than 20% of the values between strains are indicated with stronger color in the dot plot, and transparent for categories with smaller differences. The GenBank accession numbers for the genome sequences of strain Hal144^T^ and *Maribacter aestuarii* JCM 18631^T^ are CP107030 and CP107031, respectively. The general genomic features were determined using Quast 5.2 (Gurevich et al. 2013), Prokka 1.3 (Seemann 2014), and CheckM (Parks et al. 2015). The average nucleotide identities (ANI) were determined using the ANI calculator from the enveomics collection (Rodriguez-R and Konstantinidis 2016). Digital DNA-DNA hybridisation (dDDH) values were calculated using the dDDH calculator provided on the platform of the Type (Strain) Genome Server (TYGS) (Meier-Kolthoff et al. 2022). Genome-based phylogeny was calculated (Parks et al. 2022; Chaumeil et al. 2022) with strain Hal144^T^ and publicly available type strain *Maribacter* sp. genomes, applying the NJ-method, the ME-method, and the ML-method. Based on GTDBTk objective taxonomic assignments, *Maribacter litopenai* HL-LV01T (GCF_025244665.1) was omitted from genomic analysis, as its genome falls outside of the pre-defined ANI radius.

### Morphology

The morphological characteristics of strain Hal144^T^ were assessed using 7-day old cultures incubated on MB medium at 25 °C. Colony morphology and color were evaluated via observation with a magnifier, while cell morphology and motility were examined via light microscopy (Carl Zeiss Axiophot epifluorescence microscope). Gram-staining was performed using the bioMérieux Color Gram 2 Test Kit (bioMérieux Deutschland GmbH, Nürtingen, Germany) according to the manufacturer’s instructions and showed strain Hal144^T^ to be Gram-negative.

### Physiology and chemotaxonomy

The physiological and biochemical characteristics were also studied. Salinity-dependent growth was determined with 1% intervals of both, 0–7% (w/v) NaCl and 0–7% (w/v) Tropic Marine sea salt classic (Wartenberg, Germany), on a medium with the following ingredients: 5.0 g BD Bacto™ Peptone, 1.0 g BD Bacto™ Yeast Extract, 15.0 g BD Bacto™ Agar, 1 L of deionized water. The cultures were incubated at 25 °C for 7 days. Temperature-dependent growth was assessed at 5–40 °C (intervals of 5 °C) on MB for 7 days. pH-dependent growth of the strains was assessed at 5.0, 6.0, 6.5, 7.5, 8, 8.5, 9.0, and 9.5 on MB at 25 °C for 7 days with the addition of 1 M NaOH and 1 M HCl solutions to adjust the pH level. Oxygen requirements were assessed with the aerobic/anaerobic test tube method (Hogg 2013) using soft agar MB medium (7.48 g Difco™ Marine Broth 2216, 1.2 g BD Bacto™ Agar in 200 ml of deionized water) and incubation at 25 °C for 1 week. The presence of pigments was also investigated. The KOH test was performed with 7-day old cultures of the strain Hal144^T^ to detect the presence of flexirubin-type pigments (Bernardet et al. 2002). 3% (v/v) hydrogen peroxide was added to colonies of the strains and the formation of gas bubbles (Iwase et al. 2013) was observed to determine catalase activity. Oxidase activity was tested by smearing colonies onto a non-impregnated filter paper disc soaked with bioMérieux oxidase reagent (N,N,N,N-tetramethyl-1,4-phenylenediamine) and observing the development of a violet to purple coloration within 10–30 s according to the manufacturer’s instructions.

Specific enzymatic activities were studied using the semi-quantitative API® ZYM test kit (bioMérieux) according to the manufacturer’s instructions using 0.9% NaCl solution as an inoculum. The test strips were incubated for a period of 18 h at 25 °C in the dark. Growth of strain Hal144^T^ was assessed at 25 °C on MB agar plates in comparison to liquid MB medium (100 ml MB in 300 ml Erlenmeyer flasks with three baffles, 120 g).

Cellular fatty acids of strain Hal144^T^ were analysed by DSMZ Services (Leibniz Institute DSMZ, Braunschweig, Germany) using a 7-day old culture grown on MB medium at 25 °C. Briefly, fatty acid methyl esters were obtained by saponification, methylation, and extraction using minor modifications of the methods of Miller et al*. *(1982) and Kuykendall et al. (1988). The fatty acid methyl mixture was separated using a device consisting of an Agilent 7890B gas chromatograph fitted with a 5% phenyl-methyl silicone capillary column (0.2 mm × 25 m), a flame ionization detector, an Agilent model 7683A automatic sampler, and a HP-computer with MIDI data base (Hewlett-Packard Co., Palo Alto, California, U.S.A.). The Sherlock Microbial Identification System (MIS) Standard Software (Microbial ID, MIDI Labs inc, Newark, Delaware, U.S.A) automatically integrated the peaks, identified the fatty acids, and calculated their percentage content using the TSBA6 database.

The sensitivity/resistance to 29 antibiotics was tested using the disc diffusion method (Briggs and Fratamico 1999). The test was performed with Oxoid antimicrobial susceptibility test discs (Otto Nordwald GmbH, Hamburg, Germany) on MB medium, which was inoculated with a 7-day culture of strain Hal144^T^ using a swab (bioMérieux). In addition, the effect of trophodithietic acid (TDA) on the growth of strain Hal144^T^ was determined, since the compound exhibited antimicrobial activity against clinical pathogens, while TDA resistance was observed in marine bacterial isolates belonging to different taxa, including the genus *Maribacter *(Harrington et al. 2014). TDA (AdipoGen Life Sciences, Fuellinsdorf, Switzerland) was disssolved in methanol, dropped on antibiotic test discs (Ø 6 mm, Machery-Nagel, Düren, Germany), and the methanol was evaporated before placing the test disc on the culture. Plates were incubated for 7 days at 25 °C. The presence of a clear inhibition zone around the test disc indicated the susceptibility to the tested antibiotic.

## Results and discussion

### 16S and whole-genome phylogeny

**Fig. 1:**
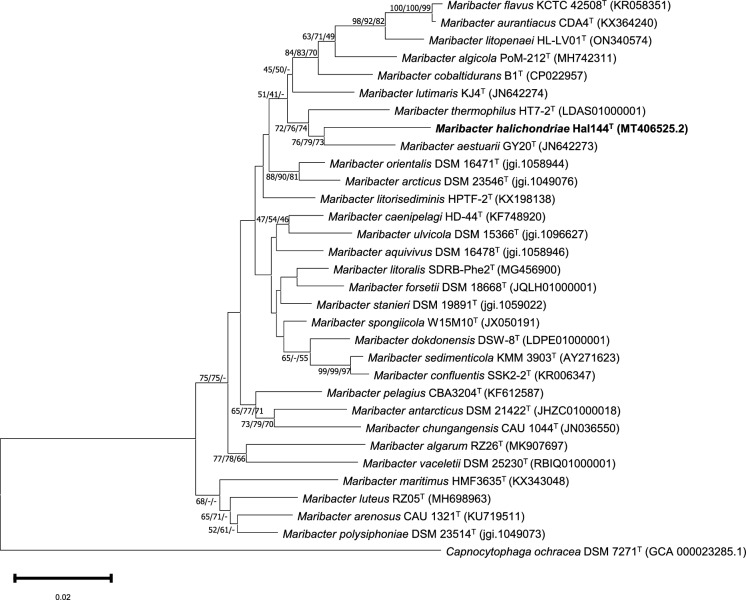
Phylogenetic relationships of Hal144^T^ based on 16S rRNA gene sequences using the Neighbor-Joining method. Bootstrap values (≥ 50%) based on 1000 replications are shown next to the branches (NJ/ME/ML). A total of 1540 positions were in the final dataset. *Capnocytophaga ochracea* DSM 7271^T^ was used as an outgroup. Bootstrap values (≥ 50%) based on 1000 replications are shown next to the branches (NJ/ME/ML). Bar, 0.02 substitutions per nucleotide position

Phylogenetic 16S rRNA gene sequence analysis revealed that the strain Hal144^T^ affiliated to the genus *Maribacter*. Applying the NJ (Fig. 1), ME, and ML method, strain Hal144^T^ clustered with *Maribacter aestuarii* GY20^T^ (= JCM 18631^T^) (96.57%). This cluster branched with *Maribacter thermophilus* HT7-2^T^ (96.31%). 16S rRNA gene sequence similarity to all *Maribacter* sp. type strains is in the range from 94.26 to 96.57% indicating that strain Hal144^T^ belongs to a new species according to a < 98.7% threshold (Chun et al. 2018).

**Fig. 2:**
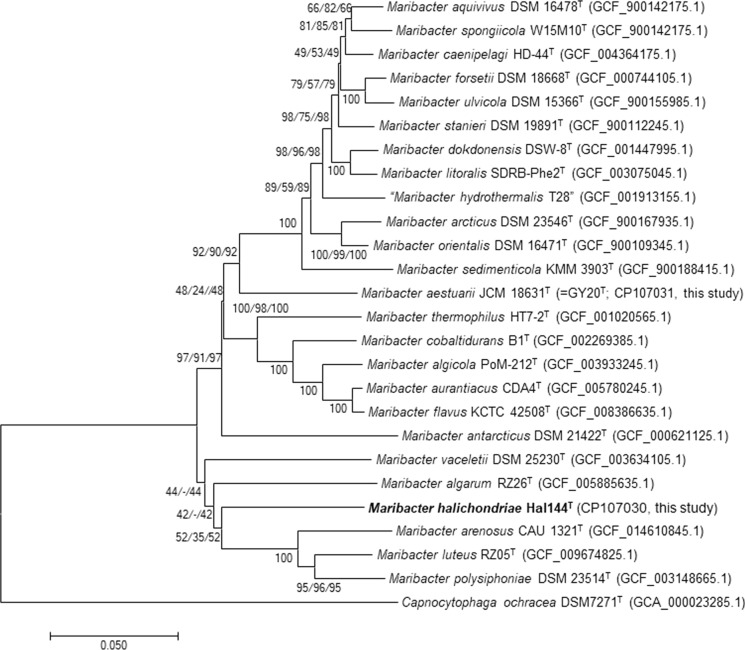
Genome phylogeny of strain Hal144^T^ was inferred using the GTDBtk pipeline. The pipeline was used with its version 2.1.0 and is based on 317,542 reference genomes. For bacterial genomes, the taxonomic identification is based on 120 single copy marker proteins. The pipeline employs MEGA version 11.0.13 to calculate the phylogenetic trees using the Neighbor Joining method. Bootstrap values (≥ 50%) based on 1000 replications are shown next to the branches (NJ/ME/ML). Bar, 0.05 substitutions per nucleotide position

Based on whole-genome phylogeny, two *Maribacter* spp. clusters branched from the outgroup (Fig. 2). Applying the NJ-method (Fig. 2), the ME-method, and the ML-method one cluster contained *Maribacter algarum* RZ26^T^, an isolate from the red alga *Gelidium amansii*, *Maribacter vaceletii* W13M1A^T^, an isolate from the sponge *Suberites carnosus*, and also strain Hal144^T^. This cluster is separated from the clade with *Maribacter luteus, Maribacter arenosus,* and *Maribacter polysiphoniae.* The differences in the 16S rRNA gene sequence phylogeny and the genome-based phylogenetic trees might be a result of the various target proteins used for the calculations, i.e. one 16S rRNA gene versus 120 single copy marker genes. Further, the different numbers of available sequences for the type strains, i.e. 25 genome sequences versus 32 16S rRNA gene sequences, may have led to divergent phylogenies. It is expected, that the comparison of phylogenetic trees will become more meaningful, when more genomic data are available for diverse *Maribacter* sp. type strains.

### Genomic characterisation

DNA G + C content was 41.4 mol% (Table 1), which is in the range of 35–41.8% as it was calculated for all 29 *Maribacter* spp. type strains with Quast in this study. The range 35–39% given in the description of the genus *Maribacter* (Parte et al. 2010) is based only on 8 *Maribacter* species. ANI values between strain Hal144^T^ and *Maribacter* sp. type strains were in the range of 75–76%. Since these mean identities are below the threshold (95–96%) for species delineation (Goris et al. 2007; Richter and Rosselló-Móra 2009), strain Hal144^T^ represents a novel species.

**Table 1:** Comparison of the general genomic features of strain Hal144^T^ and related species of the genus *Maribacter*, generated by BUSCO, CheckM and Bakta

Genome feature	1	2	3	4	5	6
Size (Mb)	4.52	3.86	4.05	4.17	4.65	5.13
G + C content (%)	41.44	39.33	38.93	39.88	38.94	40.3
N50 (bp)	4524 k	3863 k	1358 k	655 k	445 k	478 k
Completeness (%)	98.2	99.23	99.67	99.01	99.34	99.67
Contamination (%)	0.38	0.75	0.05	0.89	1.51	1.85
Number of Contigs (> 500 bp)	1	1	7	24	30	30
Coding sequences	4517	3611	3530	3742	3938	4461
Coding density	90.7	91.4	91.6	89.9	90.3	89.9
Pseudogenes	173	44	3	0	1	0
tRNA	38	41	40	37	38	40
rRNA	6	6	6	3	3	6

Strains: 1, Hal144^T^ (CP107030, this study); 2, *Maribacter aestuarii* JCM 18631^T^ (CP107031, this study); 3, *Maribacter thermophilus* HT7-2^T^ (GCF_001020565.1), [46]); 4, *Maribacter arenosus* CAU 1321^T^ (GCF_014610845.1); 5, *Maribacter luteus* RZ05^T^ (GCF_009674825.1); 6, *Maribacter polysiphoniae* DSM 23514^T^ (GCF_003148665.1)

The mean dDDH values determined for strain Hal144^T^ compared to type strains of the genus *Maribacter* were in the range of 13–13.4%, all below the suggested boundary (< 70%) for species delineation (Meier-Kolthoff et al. 2013), demonstrating that strain Hal144^T^ represents a novel genomic species.

Pseudogenes, genes with coding sequence malformations, were uniformly detected with the Bakta pipeline in strain Hal144^T^ and 5 closely related *Maribacter* sp. genomes with varying abundance (Table 1). Strain Hal144^T^ contained 3.93–173 times more pseudogenes (total of 173) compared to other related genomes, most of which were identified to contain frameshifts from the correct reading frame. Bacterial genomes often undergo pseudogenization due to changes of niche–evident as adaptations to new habitats, association with eukaryotic hosts and host-specialization (Goodhead and Darby 2015). The higher number of pseudogenes could occur due to gene redundancy caused by adapting to the symbiotic lifestyle within a microbial community specific to the sponge *H. panicea*. Future experiments should explore whether genome streamlining is present in closely related strains isolated from marine sponges.

**Fig. 3:**
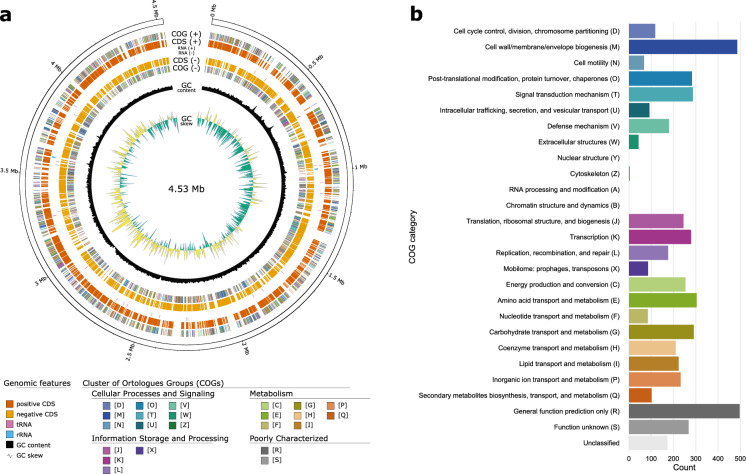
**a** Circular genome map of *Maribacter* sp. strain Hal144^T^ depicting from the outside to the center: genome coordinates, CDS on forward strand with COG category annotation, tRNA and rRNA, CDS on reverse strand with COG category annotation, GC content, and GC skew. **b** COG profile of the *Maribacter* sp. strain Hal144^T^ genome

**Fig. 4:**
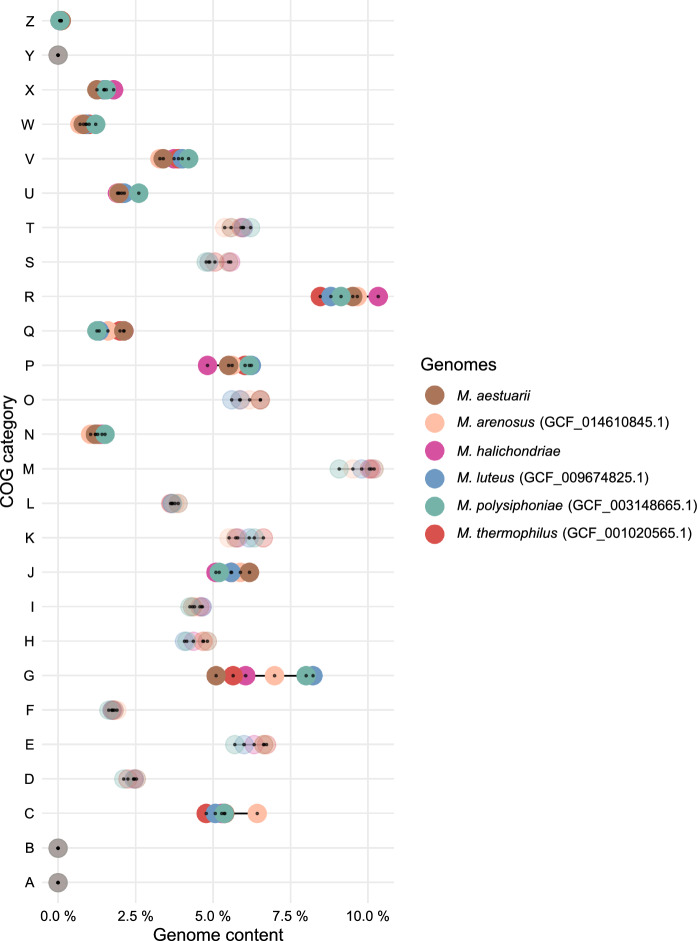
Comparative analysis of COG genome content in *Maribacter* sp. strain Hal144^T^ and 5 closely related type strains. Categories where differences between strains are less than 20% are indicated with transparent colors and categories with differences greater than 20% with full colors. (**A**) RNA processing and modification; (**B**) Chromatin structure and dynamics; (**C**) Energy production and conversion; (**D**) Cell cycle control, division, chromosome partitioning; (**E**) Amino acid transport and metabolism; (**F**) Nucleotide transport and metabolism; (**G**) Carbohydrate transport and metabolism; (**H**) Coenzyme transport and metabolism; (**I**) Lipid transport and metabolism; (**J**) Translation, ribosomal structure, and biogenesis; (**K**) Transcription; (**L**) Replication, recombination, and repair; (**M**) Cell wall/membrane/envelope biogenesis; (**N**) Cell motility; (**O**) Post-translational modification, protein turnover, chaperones; (**P**) Inorganic ion transport and metabolism; (**Q**) Secondary metabolites biosynthesis, transport, and metabolism; (**R**) General function prediction only; (**S**) Function unknown; (**T**) Signal transduction mechanism; (**U**) Intracellular trafficking, secretion, and vesicular transport; (**V**) Defense mechanism; (**W**) Extracellular structures; (**X**) Mobilome: prophages, transposons; (**Y**) Nuclear structure; (**Z**) Cytoskeleton

From the predicted genes, a total of 96.53% (4812/4985) assignments into 23/26 COG functional categories were made, with the majority belonging to general function prediction (R), cell wall/membrane/envelope biogenesis (M), amino acid transport and metabolism (E), and carbohydrate transport and metabolism (G) (Fig. 3). Comparing COG profiles with the 5 closely related type strains shows comparable values (less than 20% difference between counts in two strains) in the abundant COG categories, with exception of category R and G. In the comparison, strain Hal144^T^ contains the highest number of genes belonging to the poorly characterised COG category (R), which could be an indication of functional novelty not yet captured or defined in reference database, or artifacts from long read-only genome assembly generating more indel errors, than short-read assemblies, resulting in the fragmentation of genes and associated protein domains into multiple coding sequences. Compared to the other strains, Hal144^T^ also contains the lowest number of genes assigned to the inorganic ion transport and metabolism category (P)–lacking proteins related to the transport and exchange of sodium and calcium/hydrogen/sulfate (Fig. 4). Marine bacteria are known to exhibit a high specificity for sodium in order to maintain the cell stability in a saline medium, induce growth, and cotransport metabolites (Drapeau et al. 1966). Since bacteria are hosted extracellularly inside the sponge mesohyl matrix, the lower number of transport proteins specifically for sodium in Hal144^T^ compared to its free-living marine relatives, could indicate an adaptation to a medium physically different from ambient seawater. Alternatively, a lower number of transport proteins is also characteristic for specialists, which could confirm the highly specialized nature of this bacterium to the sponge it was isolated from (Ren and Paulsen 2005).

The functional potential unique to strain Hal144^T^ was identified by annotating the genomes with the NCBI-PGAP (Tatusova et al. 2016) pipeline, their proteins further functionally annotated based on orthology assignments in eggNOG (Huerta-Cepas et al. 2019), and mapped to higher-level functions (pathways, modules) in the KEGG database (Kanehisa and Goto 2000). Based on the selective presence of metabolic features, several protein functions (Table 2), one metabolic pathway and two reaction modules were identified. The phosphoenolpyruvate (PEP)-dependent phosphotransferase system (PTS) pathway, enabling the uptake of specific carbohydrates (e.g. fructose) with PEP as an energy source, was identified to be unique to strain Hal144^T^ based on the absence of 4 proteins (fruAb, fruA, ptsH, and ptsI) in the other genomes. The Kdo2-lipid A modification pathway module, facilitating the modification of lipopolysaccharides in Gram-negative bacteria in response to environmental stimuli, was specific to strain Hal144^T^ based on the selective presence of a lipid A 3-O-deacylase (lpxR). Finally, the pentose phosphate pathway (fructose–6P→ =ribose 5P), for the generation of NADPH, pentoses and other precursors for the synthesis of nucleotides, based on the selective presence of a 3-hexulose-6-phosphate synthase (hxlA).

**Table 2:** Differential characteristics of strain Hal144^T^ and phylogenetically related *Maribacter* sp. type strains derived from the comparative genome analysis

Characteristic			1	2	3	4	5	6
Database	Code	Description						
BV-BCR	PGF_0041633	Small multidrug export protein (qacE)	+	+	−	−	−	−
BV-BCR	PGF_03009030	Multiple antibiotic resistance protein MarC	+	−	+	+	+	+
BV-BCR	PGF_08030842	Transcriptional regulator, ArsR family	+	−	−	−	−	−
BV-BCR	PGF_00058991	Transcriptional regulator, ArsR family	+	−	−	−	−	−
BV-BCR	PGF_08848526	Transcriptional regulatory protein zraR	+	+	+	−	+	+
RAST	None	Cobalt–zinc–cadmium resistance	+	+	−	−	−	+
BV-BCR	PGF_00423898	DsrC family protein	+	−	−	+	−	−
EggNOG	K01262,K01271	Creatinase*/Prolidase N-terminal domain (Creatinase_N,Peptidase_M24), pepQ	+	−	−	−	−	−
KEGG	K02769	fructose PTS system EIIB component [EC:2.7.1.202], fruAb	+	−	−	−	−	−
KEGG	K02770	Fructose PTS system EIIBC or EIIC component [EC:2.7.1.202], fruA	+	−	−	−	−	−
KEGG	K02784	Phosphocarrier protein HPr, ptsH	+	−	−	−	−	−
KEGG	K08483	Phosphoenolpyruvate-protein phosphotransferase (PTS system enzyme I) [EC:2.7.3.9], ptsI	+	−	−	−	−	−
KEGG	K09953	lipid A 3-O-deacylase [EC:3.1.1.-], lpxR	+	−	−	−	−	−
KEGG	K08093	3-hexulose-6-phosphate synthase [EC:4.1.2.43], hxlA	+	−	−	−	−	−
EggNOG	K11031	Thiol-activated cytolysin	+	+	+	−	+	−
EggNOG	K01667	Tryptophanase, tnaA	+	−	−	−	−	−
EggNOG	K01420	Crp/Fnr family transcriptional regulator	+	−	+	−	−	−
RAST	None	Chitin and N-acetylglucosamine utilization	+	−	−	−	−	+
RAST	None	Arabinose metabolic pathway	−	−	+	+	+	−
RAST	None	Serine-glyoxylate cycle	+	−	−	−	−	−
RAST	None	Hemin transport system (ferric siderophore transport system)	+	−	+	−	+	−
antiSMASH	None	Flexirubin**	+	−	+	−	−	−

Strains: 1, Hal144^T^ (CP107030, this study); 2, *Maribacter aestuarii* JCM 18631^T^ (CP107031, this study); 3, *Maribacter thermophilus* HT7-2^T^ (GCF_001020565.1); 4, *Maribacter arenosus* CAU 1321^T^ (GCF_014610845.1); 5, *Maribacter luteus* RZ05^T^ (GCF_009674825.1); 6, *Maribacter polysiphoniae* DSM 23514^T^ (GCF_003148665.1)

*Related *Maribacter* spp. genomes have aminopeptidase P (AMP_N) (M24), similar to the creatinase N-terminal domain

**AntiSMASH analyses and biochemical pigment-assays revealed same results

Chitin, a polymer of N-acetyl-β-D-glucosamine (GlcNAc), is the most abundant biopolymer in the marine environment (Rinaudo 2006) and is an important structural component within the structural fibers of sponges belonging to the class Demospongiae (Wysokowski et al. 2013). The host sponge *H. panicea* also contains chitin, which could potentially be cleaved by sponge-associated bacteria with endo- and exo-chitinases into oligomers and dimers (Raimundo et al. 2021). Hal144^T^ is able to produce the monomer GlcNAc by its N-acetyl-β-glucosaminidase activity. RAST–server (Aziz et al. 2008) and web-resources of the Bacterial and Viral Bioinformatics Resource Center (BV-BRC) (Olson et al. 2023) were used to prove the presence of enzymes and transporters involved in the utilization of GlcNAc in strain Hal144^T^. GlcNAc from the environment might be transferred into the periplasm by an outer membrane protein (OmpA). The following four transport systems for GlcNAc from the periplasma to the cytoplasma were identified: (i) N-acetylglucosamine-specific phosphotransferase system (EC 2.7.1.69) consisting of the IIA (NagEa), IIB (NagEb), and IIC (NagEc) component, (ii) N-acetylglucosamine transporter (NagP), (iii) N-acetylglucosamine related transporter (NagX), and (iv) ATP-binding cassette (ABC) N-acetyl-D-glucosamine transporter system consisting of ATP-binding protein (ABCa), permease protein 1 (ABCb1), permease protein 2 (ABCb2), and sugar-binding protein (ABCc). The NagE system releases GlcNAc-6P. Acetate is cleaved by N-acetylglucosamine-6-phosphate deacetylase (NagA, EC 3.5.1.25) and glucosamine-6-phosphate deaminase (NagB1 and NagB2, EC 3.5.99.6) metabolizes glucosamine-6P to fructose-6P by cleaving ammonia. Fructose-6P is further processed in the glycolysis.

The genome of strain Hal144^T^ contains genes coding for enzymes (EC 2.1.2.1 serine ↔ glycine, EC 2.6.1.44 glycine ↔ glyoxylate, EC 2.6.1.51 serine → hydroxypyruvate → … → glyoxylate metabolism) involved in the serine-glyoxylate cycle. This metabolic cycle fulfils the carbon needs of bacteria in case sugars such as glucose are not available.

The Gram-negative strain Hal144^T^ exhibited resistance against the antibiotics ampicillin, bacitracin, mupirocin, and oleandomycin, which are mainly used against Gram-positive bacterial infections. Resistance to antibiotics against Gram-negative bacteria was observed. Among them were all five aminoglycosides tested in this study i.e. amikacin, kanamycin, gentamicin, neomycin, and streptomycin, and the polypeptide polymyxin B. Membrane-associated antibiotic resistance, e.g. by the expression of multidrug efflux pumps, is a key mechanism in Gram-negative bacteria (Du et al. 2018; Davin-Regli et al. 2021). Therefore, genome analysis focused on related characteristics was performed applying the RAST–server (Aziz et al. 2008), and two multidrug resistance efflux pumps families were detected. One efflux pump system belongs to the multi antimicrobial extrusion protein (MATE), a Na^+^/drug antiporter. The second system, called resistance nodulation division (RND), a H^+^/drug antiporter, confers resistance to antimicrobial compounds produced by the host and plays a role in colonization, persistence, and dissemination of bacteria in the host (Du et al. 2018). In addition, a gene coding for CmeC, an outer membrane channel protein originally described in efflux systems from *Campylobacter* sp. strains (Davin-Regli et al. 2021), was found.

Since there are only two publications available, which include information on genome analysis of a *Maribacter* sp. type strains (Hu et al. 2015b; Kim et al. 2023), we displayed genome-derived features of strain Hal144^T^ in comparison to the next related type strains in Table 2. Genomic information of strain Hal144^T^ support differentiation from the five closely related *Maribacter* spp. Among further features, serine-glyoxylate could only be predicted for Hal144^T^. Chitin and N-acetylglucosamine utilisation was shown for Hal144^T^ and *M. polysiphoniae* DSM 23514^T^, but not for the further four strains. Hal144^T^ produced the pigment flexirubin like *M. thermophilus* HT7-2T^T^ in contrast to the other four strains. Genome analysis of *M. thermophilus* HT7-2^T^ (Hu et al. 2015b) revealed the presence of genes which were also shown for Hal144^T^, i.e. oxygen-regulating Crp/Fnr proteins, heat and cold shock proteins, and two systems playing a role in heavy-metal resistance, resistance-nodulation-cell division (RND) proteins and a lead, cadmium, zinc, and mercury transporting ATPase. A second known genome analysis was carried out for *Maribacter litopenaei* HL-LV01^T^ (Kim et al. 2023). This motile strain encoded gliding motility-associated proteins, in contrast to the non-motile strain Hal144^T^. Biosynthesis genes for flexirubin and carotenoids are abundant in both strains.

### Morphology, physiology and chemotaxonomy

**Fig. 5:**
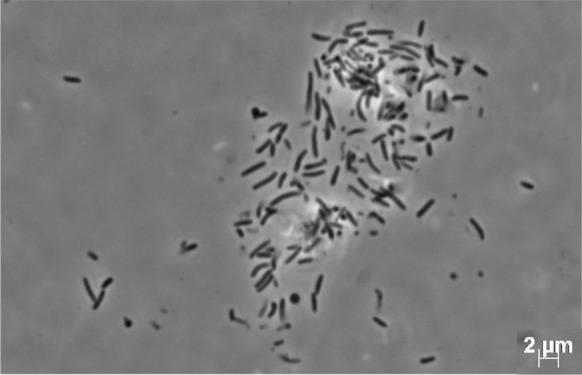
Micrograph of strain Hal144^T^ after cultivation on MB medium for 15 days at 25 °C

Cells of strain Hal144^T^ are Gram-negative, strictly aerobic, non-motile, stabs or slightly curved stabs, 0.8 µm wide and 2 µm long (Fig. 5). Colony morphology, growth conditions regarding salinity, temperature, and pH are displayed in the species description. Only with a high amount of inoculum (approximately a half culture agar plate) was growth observed in liquid media. The bacterial cells were not homogeneously distributed in liquid media. Instead, the cells formed crumbs, which attached ring-shaped on the glass wall in the aerated zone. It is assumed, that strain Hal144^T^ prefers surfaces for growth, at least when subjected to the cultivation conditions in our study. In addition, the most abundant COG group M related to cell wall/membrane/envelope biogenesis effecting production of extracellular material and biofilms. These findings could indicate, that strain Hal144^T^ contributes to the formation of microbial biofilms in its host *Halichondria panicea* and thus might play a role in the complex cellular dialogue of the sponge holobiont (Schmittmann et al. 2020).

Strain Hal144^T^ was positive for oxidase, catalase, alkaline phosphatase, esterase (C4), esterase lipase (C8), lipase (C14), leucine arylamidase, valine arylamidase, cystine arylamidase, α-chymotrypsin, acid phosphatase, naphthol-AS-BI-phosphohydrolase, and N-acetyl-β-glucosaminidase, and weak positive for β-galactosidase, α-glucosidase, β-glucosidase, α-mannosidase, and trypsin, but negative for α-galactosidase, β-glucuronidase, and α-fucosidase. The pigment flexirubin is produced.

The major fatty acids observed were iso-C_17_:_0_ 3-OH (25%), iso-C_15_:_0_ (25%), and iso-C_15:1_ G (14%), followed by fatty acids from the category “summed feature 3” (12%) consisting of C_16:1_ω6c and/or C_16:1_ω7c. Further fatty acids were C_14:0_ (1.1%), C_16:0_ 2.2%), anteiso-C_15:0_ (1.6%), iso-C_16:0_ (0.5%), C_15:1_ω6c (1.5%), C_17:1_ω6c 1.3%), C_18:1_ω6c (1.1%), C_15:0_ 3-OH (1.0%), C_16:0_ 3-OH (3.0%), iso-C_15:0_ 3-OH (4.8%), iso-C_16:0_ 3-OH (1.9%), and summed feature 9″ consisting of iso-C_17:1_ω9c and/or C_16:0_ 10-methyl. The overall fatty acid pattern of strain Hal144^T^ was similar to those described for other *Maribacter* sp. type strains (Parte et al. 2010). In contrast to *M. aestuarii* JCM 18631^T^, *M. arenosus* CAU 1321^T^, *M. luteus* RU05^T^, and *M. polysiphoniae* DSM 23514^T^, strain Hal144^T^ did not produce C_17:1_ω8c (Table 3). The hydroxy fatty acids C_15:0_ 3-OH and C_16:0_ 3-OH were shown for strain Hal144^T^, but not for *M. thermophilus* HT7-2^ T^.

**Table 3:** Selected differential phenotypic characteristics of strain Hal144^T^ and phylogenetically related *Maribacter* sp. type strains

Characteristic	1	2	3	4	5	6
Origin	Sponge	Sediment	Alga	Sediment	Sand	Alga
Colony colour	Y	YO	Y	Y	Y	Y
Gliding motility	−	+	+	−	+	+
Fatty acids not present at Hal144^T^	−	C_17: 1_ω8c	C_15: 0_	C_17: 1_ω8c	C_17: 1_ω8c	C_15: 0_
	−	iso-C_16: 1_ H	unknown ECL13.565	C_17: 0_ 3-OH	C_17: 0_ 3-OH	C_17: 1_ω8c
*Growth conditions*						
Temperature range (°C)	5–30	10–30	4–50	20–30	7–40	4–41
Temperature optimum (°C)	25–30	25	40–42	30	30	30–32
pH range	5.0–8.0	6.5–10.5	5.5–8.8	6.5–9.5	5.5–9.0	5.5–10.0
pH range	6.5–7.5	7.0–8.0	7.0	8.0	7.0	7.5–8.5
Sea salt range (% w/v)	2.0–6.0	nd	0.5–10.0	nd	nd	nd
Sea salt optimum (% w/v)	3.0–4.0	nd	2.5	nd	nd	nd
Enzyme activities (API ZYM)						
α-Chymotrypsin	+	+	( +)	−	+	+
α-Fucosidase	−	−	−	−	+	−
Lipase (C14)	+	−	−	−	( +)	nd
Production of flexirubin	+	−	+	−	−	−

Strains: 1, Hal144^T^; 2, *Maribacter aestuarii* JCM 18631^T^; 3, *Maribacter thermophilus* HT7-2^T^; 4, *Maribacter arenosus* CAU 1321^T^; 5, *Maribacter luteus* RZ05^T^; 6, *Maribacter polysiphoniae* DSM 23514^T^. Data for taxa 2, 3, 4, 5, and 6 from Lo et al. (2013), Hu et al. (2015a, b), Thongphrom et al. (2016), Liu et al. (2020), and Nedashkovskaya et al. (2007), respectively. Y, yellow; YO, yellow-orange; +, positive; −, negative; (+), weakly positive; nd, not determined

Strain Hal144^T^ displays sensitivity to cefoxitin (30 µg), chloramphenicol (50 µg), ciprofloxacin (5 µg), doripenem (10 µg), doxycycline (30 µg), imipenem (10 µg), linezolid (30 µg), norfloxacin (10 µg), novobiocin (30 µg), ofloxacin (5 µg), rifampicin (30 µg), teicoplanin (30 µg), tetracycline (30 µg), and vancomycin (30 µg). Exhibits resistance to amikacin (30 µg), ampicillin (10 µg), bacitracin (10 units), kanamycin (30 µg), mupirocin (200 units), gentamicin (30 µg), neomycin (30 µg), oleandomycin (15 µg), polymyxin B (300 units), streptomycin (25 µg), and trophodithietic acid (2 µg). Variable reactions for erythromycin (15 µg), nalidixic acid (30 µg), lincomycin (15 µg), oxacillin (5 µg), and penicillin G (10 units).

In addition to characteristics obtained from genome analyses (Tables 1, 2), strain Hal144^T^ can also be differentiated from phylogenetically related type strains based on phenotypic features (Table 3). Strain Hal144^T^ showed non-gliding motility, a characteristic shared with *M. arenosus* CAU 1321^T^, but not with the further four type strains. Enzyme activities such as α-chymotrypsin and lipase (C14) were exhibited by strain Hal144^T^, but not by *M. arenosus* CAU 1321^T^. Flexirubin was produced by strain Hal144^T^ and *M. thermophilus* HT7-2^T^ in contrast to the other type strains. The temperature range of strain Hal144^T^ was 5–30 °C which differentiated the strain from *M. thermophilus* HT7-2^T^ growing in the range of 4–50 °C.

## Conclusion

Based on genotypic and phenotypic features of the strain Hal144^T^ a novel species of *Maribacter* was described, for which the name *Maribacter halichondriae* is proposed. Hal144^T^ exhibited features that point towards a lifestyle in the sponge environment, such as a high number of pseudogenes, low number of genes related to the transport and exchange of sodium, and contained genes related to biofilm production, N-acetyl-β-D-glucosamine utilisation, and antimicrobial resistance.

### Description of *Maribacter halichondriae* sp. nov.

*Maribacter halichondriae* (ha.li.chon'dri.ae. N.L. gen. n. *halichondriae*, of the sponge genus *Halichondria*).

Cells are Gram-negative, strictly aerobic, non-motile, stabs or slightly curved stabs, 0.8 µm wide and 2 µm long. Colonies are circular, raised, orange, and brittle, 1–2 mm in diameter. Growth occurs on 2–6% (w/v) sea salt (optimum 3–4%), no growth on NaCl as the sole salt supplement, at 5–30 °C (optimum 25–30 °C), and at pH 5.0–8.0 (optimum pH 6.5–7.5). Strain is oxidase- and catalase-positive. Utilise N-acetylglucosamine. Flexirubin-type pigment is present. The major fatty acids (> 5% of total composition) are C_17_:_0_ 3-OH, iso-C_15_:_0_, and iso-C_15:1_ G. The DNA G + C content of Hal144^T^ is 41.4 mol%.

The type strain Hal144^T^ (= DSM 114563^ T^ = LMG 32744^T^) was isolated from the marine sponge *Halichondria panicea* collected at Schilksee along the Kiel-Fjord of the Baltic Sea (latitude 54.424705, longitude 10.175133).

## Abbreviations

ABC: ATP-binding cassette
BCCM/LMG: Belgian Coordinated Collections of Microorganisms
BSW: Baltic Sea water
DSMZ: Leibniz-Institut DSMZ-Deutsche Sammlung von Mikroorganismen und Zellkulturen
GlcNAc: N-acetyl-β-D-glucosamine
MB: Marine medium
g: Centrifugal force
